# NF-κB in Innate Neuroprotection and Age-Related Neurodegenerative Diseases

**DOI:** 10.3389/fneur.2015.00098

**Published:** 2015-05-20

**Authors:** Annamaria Lanzillotta, Vanessa Porrini, Arianna Bellucci, Marina Benarese, Caterina Branca, Edoardo Parrella, Pier Franco Spano, Marina Pizzi

**Affiliations:** ^1^Department of Molecular and Translational Medicine, National Institute of Neuroscience, University of Brescia, Brescia, Italy; ^2^IRCCS, San Camillo Hospital, Venice, Italy

**Keywords:** NF-κB, epigenetic drugs, BDNF, c-Rel deficient mice, RelA (K310)

## Abstract

NF-κB factors are cardinal transcriptional regulators of inflammation and apoptosis, involved in the brain programing of systemic aging and in brain damage. The composition of NF-κB active dimers and epigenetic mechanisms modulating histone acetylation, finely condition neuronal resilience to brain insults. In stroke models, the activation of NF-κB/c-Rel promotes neuroprotective effects by transcription of specific anti-apoptotic genes. Conversely, aberrant activation of NF-κB/RelA showing reduced level of total acetylation, but site-specific acetylation on lysine 310, triggers the expression of pro-apoptotic genes. Constitutive knockout of c-Rel shatters the resilience of substantia nigra (SN) dopaminergic (DA) neurons to aging and induces a parkinsonian like pathology in mice. c-rel^−/−^ mice show increased level of aberrantly acetylated RelA in the basal ganglia, neuroinflammation, accumulation of alpha-synuclein, and iron. Moreover, they develop motor deficits responsive to l-DOPA treatment and associated with loss of DA neurons in the SN. Here, we discuss the effect of unbalanced activation of RelA and c-Rel during aging and propose novel challenges for the development of therapeutic strategies in neurodegenerative diseases.

## Introduction

In the central nervous system, NF-κB transcription factor acts as a pleiotropic regulator of target genes controlling physiological function (1) as well as pathological processes associated with neurodegeneration (2, 3).

The NF-κB family of transcription factors is composed by five different members that are p65 (RelA), RelB, c-Rel, p50/p105 (NF-κB1), and p52/p100 (NF-κB2). The RelA subunit, composing the activated p50/RelA dimer, and its post-transcriptional modifications play a pivotal role in the onset of neurodegenerative processes triggered by ischemic insults as well as glutamate or beta-amyloid toxicity (4–8). The c-Rel subunit within activated NF-κB dimers counteracts the ischemic injury acting as an innate mechanism of neuroprotection (9). The c-Rel factor is reduced in neurons exposed to oxygen–glucose deprivation (OGD), and its over-expression can limit the cell death. Moreover, the deficiency of c-Rel induces an age-related behavioral parkinsonism in mice, with degeneration of nigral dopaminergic (DA) neurons and development of a Parkinson’s disease (PD)-like neuropathology (10). Recent evidence has shown that activation of NF-κB drives the systemic and brain aging process in mice (11, 12). Notably, Tilstra and colleagues demonstrated that RelA is the most contributing subunit in degenerative changes associated with senescence in a progeroid mice model (13).

We propose that while RelA activation accompanies normal brain aging, a misbalance between RelA and c-Rel might drive pathological aging by affecting the survival of substantia nigra (SN) DA cell and turning old mice into a parkinsonian phenotype.

## RelA and c-Rel: Two Opposing Regulators of Neuronal Resilience to Brain Ischemia

In the central nervous system, NF-κB factors are key players of a number of physiological processes such as neurogenesis (14), neuritogenesis (15), synaptic plasticity, learning, and memory (16–18). In recent years, a body of data has shown that NF-κB dysregulation participates to neurodegenerative mechanisms that occur in brain exposed to trauma or ischemia (19, 20), as well as in the brain of patients affected by PD (21, 22) and Alzheimer’s disease (23).

**Figure 1:**
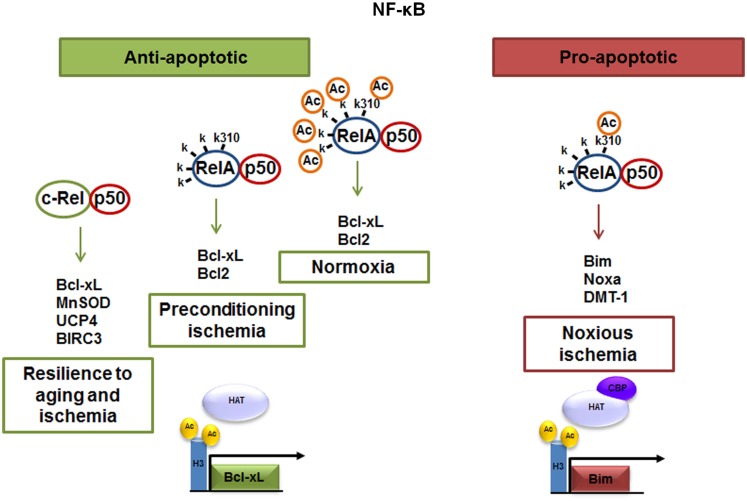
**The p50/RelA and p50/c-Rel dimers regulate neuronal survival**. Anti-apoptotic effects of NF-κB can be mediated by c-Rel containing dimers, which enhance neuronal resilience to oxidative stress by inducing Bcl-xL, MnSOD, UCP4, and BIRC3 expression. NF-κB anti-apoptotic effects can also be mediated by p50/RelA during preconditioning ischemia through the transcription of Bcl-xL. The pro-apoptotic effect elicited by NF-κB p50/RelA dimer in noxious ischemia is dependent on changes in the RelA acetylation state. A lower grade of RelA general acetylation, respect to control condition, but a site-specific acetylation on Lys 310 residue addresses the NF-κB binding toward Bim, Noxa, and DMT1 promoter.

The neuronal response to external stimuli relies on a differential activation of NF-κB dimers. We found that targeting RelA or c-Rel expression by antisense oligonucleotides (5) or siRNAs (6, 9) produces opposite effects on neuron survival. While over-activated p50/RelA dimers contribute to the apoptotic program, the c-Rel containing dimers increase the resilience of injured neuronal cells (Figure 1). Neurotoxic stimuli, such as ischemia (4, 9), glutamate (5), β-amyloid (7, 24), or 1-methyl-4-phenylpyridinium (MPP^+^) (25, 26), induce p50/RelA dimers activation and the transcription of a panel of pro-apoptotic genes (4). Conversely, c-Rel-containing dimers are responsible for anti-apoptotic gene expression by signals promoting neuroprotection in diverse neurotoxic settings, such as S100B in models of NMDA-mediated excitotoxicity (27), agonists at mGlu5 receptors against β-amyloid- (7) and MPP^+^ toxicity (25) or adipocyte-derived hormone leptin in neurons exposed to OGD (28). Over-expression of c-Rel in cultured neurons promotes anti-apoptotic effects by inducing the transcription of manganese superoxide dismutase (MnSOD) and Bcl-xL (7, 29, 30). c-Rel overabundance also limits the generation of reactive oxygen species (ROS) by inducing transcription of the mitochondrial uncoupling proteins 4 (UCP4) (31), a brain-specific mitochondrial ion channel producing mild reduction of mitochondrial membrane potential and neuroprotection (32). Moreover, c-Rel can control the expression of baculoviral IAP repeat-containing protein 3 (BIRC3), named also as cIAP2, an anti-apoptotic E3 ligase, which can modulate the receptor interacting protein 1 (RIP1) activity by ubiquitination (33). Depending on its ubiquitination status, RIP1 can dictates if tumor necrosis factor (TNF)-α induces cell survival (and inflammation), or cell death pathways (34). Ubiquitinated RIP1 can recruit other kinases and finally induce NF-κB-mediated transcription of prosurvival and pro-inflammatory genes (TNF-α-dependent NF-κB activation) (35).

The elucidation of the dual effects of NF-κB activation on neuron survival was more evident in studies of severe brain ischemia. The activation of p50/RelA rapidly occurs in neurons and glial cells and has been implicated in pathogenesis of post-ischemic injury (36–38). In brain ischemic tissue of mice subjected to permanent middle cerebral artery occlusion (MCAO) and in primary cortical neurons exposed to OGD, NF-κB followed a similar pattern of activation (8, 39) characterized by increased nuclear translocation of p50/RelA dimer (4, 36) and decreased translocation of c-Rel-containing dimers (9). In these conditions, NF-κB activity was associated with an unbalance expression of pro-apoptotic RelA target genes, i.e., an increased expression of the pro-apoptotic members of Bcl-2 family genes (4) and reduced level of the anti-apoptotic member Bcl-xL (9, 40). During brain ischemia, RelA induced the expression of the 1B isoform of the divalent metal transporter-1 (1B/DMT1), the membrane carrier responsible for iron accumulation and brain damage after injury (41). The RelA-induced 1B/DMT1 expression acted as an upstream mechanism responsible for iron accumulation and contributing to neuronal cell death. While the over-expression of RelA increased cell death, the over-expression of c-Rel prevented neuronal loss in cortical neurons exposed to OGD, by increasing the transcription of Bcl-xL gene (9, 39). Knocking-down c-Rel expression exacerbated neuronal susceptibility to OGD-mediated damage. Under brain ischemia, mice deficient for the c-Rel factor appeared insensitive to neuroprotective activity of leptin, a c-Rel inducer capable to limit cortical damage in wild-type mice (24, 28). These data strongly suggested that inhibition of c-Rel-containing dimers and activation of p50/RelA are key events in the pathogenesis of post-ischemic brain injury. In spite of these premises, p50/RelA activation *per se* appeared to be insufficient to drive pro-apoptotic transcription during brain ischemia. A similar pattern of p50/RelA nuclear translocation was found in mice exposed to a brief preconditioning ischemia (8) generating brain tolerance to a subsequent lethal ischemic injury (42). In neuronal cells, likewise in tumor cells, gene targeting by p50/RelA is finely regulated by post-transcriptional modification of RelA subunit, such as phosphorylation and acetylation (43). These modifications shape the strength and specificity of the NF-κB–DNA binding and final transcriptional responses. On this line, we investigated whether the activation of the p50/RelA dimer, in preconditioning or in lethal ischemia, differs in the RelA acetylation state (44).

## RelA Acetylation is a Dynamic Process Which Tunes p50/RelA-Mediated Pro-Apoptotic Transcription in Brain Ischemia and is Modulated by Epigenetic Drugs

Acetylation is the key post-translational modification of histones that controls the accessibility of chromatin to the transcriptional machinery and plays an essential role in gene activation (45). Lysine acetylation is reversible and controlled by the opposing activities of histone acetyltransferase (HAT) and histone deacetylase (HDAC).

Besides histones, diverse non-histone proteins, including transcription factors NF-κB, are modified by HAT co-activators and HDACs (46). Acetylation of RelA on specific lysine residues (K122, 123, 218, 221, and 310) is a dynamic process that differently affects the RelA interaction with IκBα, the DNA-binding ability and the transcriptional activity of the protein (43, 47).

Members of class I HDACs, particularly HDAC1, HDAC2, and HDAC3, inhibited by vorinostat and entinostat (MS-275) are the most responsible for the general deacetylation of RelA (43, 48). Conversely, sirtuin 1 (SIRT1), an atypical class III HDAC that requires nicotinamide adenosine dinucleotide (NAD^+^) rather than zinc as a co-factor (49), activated by resveratrol, selectively deacetylates RelA at lysine 310 (K310) residue (8, 50).

Our studies have shown that mechanisms affecting the acetylation state of RelA might discriminate between protective and neurotoxic activation of NF-κB during ischemia (8). Protective ischemic preconditioning and harmful ischemia induced similar levels of p50/RelA activation, but only the ischemic injury induced an atypical RelA acetylation. The RelA that translocated to the nucleus in primary cortical neurons exposed to preconditioning OGD, or in cortices of mice subjected to preconditioning MCAO, showed a general deacetylation that paralleled the deacetylation on the K310 residue. Conversely, RelA activated in neurons exposed to lethal OGD or in cortices of mice subjected to noxious ischemia displayed a general deacetylation, but site-specific acetylation on the K310 residue. This suggested that a mismatch between general and “site-specific” (K310) acetylation of RelA – where total acetylation decreases and K310 acetylation increases – could be responsible for pro-apoptotic transcription in ischemic conditions (8). The relevance of K310 acetylation to RelA-mediated effects during ischemia was demonstrated by mutagenesis analysis. The substitution of lysine with arginine at the RelA 310 residue impaired the acetylation at this site. In cells expressing the mutated RelA subunit, the OGD-mediated DMT1 transcription and the cell damage were totally prevented (8, 41). By undergoing such aberrant acetylation, RelA detached from the anti-apoptotic *Bcl-xL* promoter to bind the pro-apoptotic *Bim* promoter (44). In addition to changing the acetylation state of RelA, lethal ischemia produced a significant reduction of H3 histone acetylation (44), in line with previous evidence (51).

Prompted by these findings and in order to correct altered acetylation of RelA and histones after brain ischemia, we studied the association of the specific class I HDAC inhibitor MS-275 (52), and resveratrol (53). MS-275 is a synthetic benzamide derivative that currently is under clinical evaluation for cancer therapy (54). MS-275 has been shown to inhibit HDAC 1-3 with excellent pharmacokinetic properties (52).

Resveratrol is a polyphenol with multiple activities, including anti-oxidant, anti-tumorigenic, and neuroprotective activity (53, 55). In various models of brain ischemia, resveratrol delayed axonal degeneration after injury and mitigated the formation of free radical species as well as mitochondria-mediated apoptosis (56–59).

Widely known mechanisms of resveratrol action include the activation of the longevity factors SIRT1 (60) and AMP-activated kinase (AMPK), a serine–threonine kinase that acts as a key metabolic and stress sensor/effector (61). We found that treatments with either MS-275 or resveratrol in the post-ischemic period of mice subjected to MCAO decreased the infarct volume and displayed a significant neuroprotective activity in cortical neurons exposed to OGD (44). What’s more, we showed that the combination of MS-275 and resveratrol at sub-threshold doses elicited a synergistic effect leading to maximal neuroprotection in both the animal and the cellular models of brain ischemia. MS-275 at the highest concentration tested, 1 μM, increased acetylation of H3 histones on K9/18 residues in neurons exposed to OGD. Resveratrol, unable to modify *per se* the H3 acetylation, produced a synergistic acetylation of H3 K9/18 when used in combination with MS-275.

Notably, the synergistic effect produced by co-administration of low doses of MS-275 (0, 1 μM) and resveratrol (3 μM) was sustained by AMPK activation by resveratrol. This could be ascribed to the fact that AMPK can activate many catabolic pathways to produce ATP and acetyl-CoA (62), the fundamental co-factor for HAT activity. AMPK has also been found to indirectly support the resveratrol-dependent SIRT1 induction by inducing NAD^+^ generation (61). As a consequence of AMPK-mediated enhancement of HAT and SIRT1 activity, the combination of MS-275 and resveratrol reversed the mismatch of RelA acetylation state in neurons exposed to OGD by, respectively, enhancing the RelA general acetylation and by reducing the acetylation at the K310 residue. The neuroprotective effect and transcription of anti-apoptotic genes observed following the treatment with the drug combination appeared closely related to the restored optimal RelA acetylation state (8, 63). The protective and transcriptional effects produced by resveratrol and MS-275 in cortical neurons were entirely reproduced in the mouse MCAO model. The combination of sub-threshold doses of the drugs, administered during the reperfusion period, elicited a synergistic effect that limited the cerebral infarct volume and the subsequent neurological deficits. MS-275 and resveratrol in combination showed a long-lasting efficacy as the beneficial effects were still evident 72 h after the injury. Moreover, they displayed a wide therapeutic window as their efficacy was evident when administered within 7 h after the ischemic onset. The treatment induced a transcriptional switch from pro- to anti-apoptotic genes. The RelA binding shifted from the *Bim* to the *Bcl-xL* promoter and the acetylation of associated histones changed accordingly. H3 acetylation decreased at the *Bim* and increased at the *Bcl-xL* gene.

Recently, we evaluated the acetylation of histone residues at the brain-derived neurotrophic factor (BDNF) IV promoter in primary mouse cortical neurons exposed to OGD and treated with the synergistic combination of MS-275 and resveratrol (64).

We focused on promoter IV, which is known to be important for synaptic plasticity, both during neuronal development and in the adult brain (65). In the cortex, the promoter IV-dependent BDNF transcription accounts for the majority of the neuronal activity-induced BDNF expression (66, 67). Several studies have proposed BDNF as possible mediators of the beneficial effects of HDAC inhibitors in nervous system disorders (68–70). A ChIP analysis in cortical neurons showed that histones at the BDNF promoter IV were deacetylated after OGD exposure. Treatment in the post-OGD period with the combination of MS-275 and resveratrol significantly increased acetylation at H3K9/18 and H4K12 histones (64). These histone modifications may act cooperatively and possibly in parallel to other histone modifications to increase BDNF expression.

It can be proposed that neuroprotection elicited by MS-275 and resveratrol treatment is also closely related to modulation of BDNF expression and may improve neurologic function by enhancing neuronal plasticity.

All together, these data provide evidence that a pharmacological intervention targeting the epigenetic machinery can represents a promising strategy to limit post-ischemic injury with an extended therapeutic window.

## c-Rel Deficiency Causes a Progressive Late-Onset Parkinsonism in Mice

Following the evidence that RelA and c-Rel play opposing effects on neuron survival, and prevalence of p50/RelA activation versus p50/c-Rel triggers apoptotic cell death in brain ischemia (9), we tested whether a constitutive defect in c-Rel protein might affect the brain aging. Behavioral and pathological analyses of c-Rel knockout mice were performed at 2, 12, and 18 months of age and c-rel^−/−^ mice showed to develop a PD-like syndrome and pathology with aging (10). Besides c-Rel subunit, other NF-κB subunits have been previously investigated for possible correlation with the onset of a parkinsonian pathology. Increased levels of RelA have been detected in the brains of MPTP-intoxicated mice (21) as well as in the brain of subjects affected by PD (21, 22, 71). This increase was evident both in neuronal and glial cells of the SN, suggesting a role of RelA activation in neuronal cell loss and neuroinflammatory response associated with PD progression. The role of the other NF-κB subunits in PD remains unclear. The p50^−/−^ mice treated with MPTP did not behave differently from wild-type mice (72), suggesting no, or only minor, role for p50 in the regulation of SN neuron resilience.

**Figure 2:**
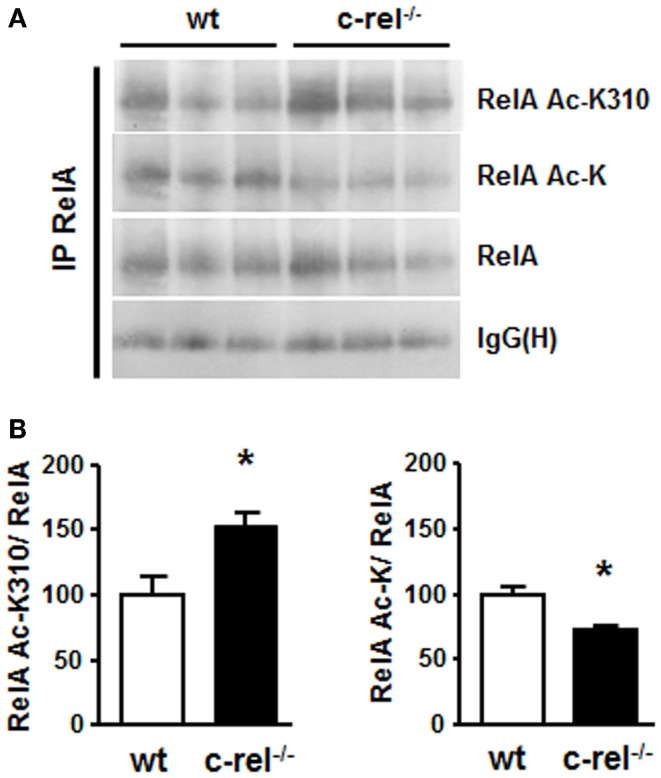
**RelA acetylation in the striatum of c-rel^−/−^ and wt mice**. **(A)** Representative picture of the immunoprecipitation analysis of RelA acetylation in total proteins of caudatus putamen. RelA acetylation at K310 residue increased, while total RelA acetylation was reduced in striatal total extracts of 18-month-old c-rel^−/−^ mice. No significant change was detected in the total RelA content. **(B)** Values from densitometry analysis of immunoblots are expressed as a percentage of the wt value. The signal given by IgG(H) is used as a control for the quality of the immunoprecipitation. Bars depict the mean ± SEM (*n* = 4 animals per group), *p < 0.01 versus wt value.

In 18-month-old c-rel^−/−^ mice, the analysis of tyrosine hydroxylase (TH)-positive cells of the SN pars compacta (SNc) revealed a loss of DA neurons that paralleled the total loss of Nissl-stained cells. No significant change in the estimated number of DA cells was evident in 2- or 12-month-old c-rel^−/−^ mice compared to age-matched controls. Notably, the loss of SNc DA neurons was associated with a decrease of TH-positive fibers and reduction of dopamine transporter (DAT) and dopamine content in the striatum. The 18-month-old c-rel^−/−^ mice displayed no significant degeneration in the other brain areas examined, the nucleus basalis magnocellularis and the medial septal area, or in the ventral tegmental area that is generally spared in PD. Additional examination of SNc in aged c-rel^−/−^ mice revealed a marked immunoreactivity for α-synuclein, the main protein constituent of Lewy bodies and Lewy neuritis and the key pathological feature of PD (73). Of note, fibrillary aggregated α-synuclein, detected by Thioflavin-S labeling, was present in the spared DA neurons of the SNc. Accumulation of insoluble α-synuclein in the mesencephalon was confirmed by the presence of a monomeric α-synuclein in the urea/SDS extracts used to solubilize the insoluble fraction. Neuroinflammation characterized by chronic microglial reactivity, RelA activation, and iron accumulation are also important features of the PD neuropathology (74, 75). As observed in PD brain (76), SNc and striatum of aged c-rel^−/−^ mice showed marked signs of microglia activation with increased number, swollen cell bodies, and thick processes of CD11b-positive cells. Both SNc and striatum of aged c-rel^−/−^ mice displayed elevated levels of iron and increase of the iron transporter DMT1 that, as mentioned above, is a transcriptional target of aberrantly acetylated RelA. Preliminary investigation on NF-κB in striatal extracts of 18-month-old c-rel^−/−^ mice confirmed, indeed, the presence of aberrantly acetylated RelA as reported in Figure 2. The immunoprecipitated RelA showed reduced level of general acetylation associated with increased site-specific acetylation at the K310 residue. These changes occurred without any significant variation in the cellular amount of RelA. This finding strongly suggests that a misbalance between c-Rel and RelA can evolve during aging in c-rel^−/−^ mice to produce changes in RelA acetylation, microglia activation, and neuronal apoptosis.

Because inflammatory and neurotoxic activation of microglia have been also reported to be triggered by acetyl-RelA (K310) (77), it is feasible that microglia activation participate to the neurodegenerative process in c-rel^−/−^ mice. The extensive analysis of the neuroinflammatory profile of c-rel^−/−^ mice along with disease progression will reveal the exact entity of this inflammatory process and the specific participation of innate and adaptive immunity.

The neurochemical changes observed in aged c-rel^−/−^ mice were also accompanied by the onset of motor deficits. A significant impairment in spontaneous motor activity was evident in c-rel^−/−^ mice at 18 months, but not in younger mice as previously shown (17, 18). Indeed, either monitored for 1 h or six consecutive days to avoid stress-related bias, 18-month-old c-rel^−/−^ mice displayed a lower locomotor activity. Furthermore, the gaiting analysis supported the presence of a locomotor dysfunction related to bradykinesia and rigidity. Noteworthy, the treatment with l-DOPA plus benserazide, a cocktail that is considered the gold standard for PD therapy, totally reversed the locomotor deficits and normalized most of the gaiting parameters.

Despite these findings, how the constitutive c-Rel deficiency can specifically affect DA neurons of SNc is still an open question (10). The selective vulnerability of SNc neurons in PD has been attributed to the peculiar “energy-demanding” physiology of these cells (78), which display enormous axonal field and impressive number of synapses for each axon (79). Moreover, during their pacemaking activity, SNc DA neurons, but not the ventral tegmental area neurons, generate autonomous action potentials by unusual engaging of L-type Ca^2+^ channels, which require subsequent activation of ATP-dependent Ca^2+^ pumps to maintain Ca^2+^ homeostasis (80). The energy production by mitochondria and endoplasmic reticulum in SNc DA neurons associates with the generation of large amounts of ROS that are constantly neutralized by anti-oxidant systems, including SODs catalases, glutathione peroxidase, and UCP4 and UCP5 (81). It can be inferred that in the absence of c-Rel a reduced expression of UCP4 (31) and MnSOD (7, 29) might enhance ROS accumulation during aging in SNc neurons (82), and synergize with reduced expression of anti-apoptotic Bcl-xL (9, 30) to affect neuronal resilience. Also, it is conceivable that mitochondria impairment associated with c-Rel deficiency may switch the acetylation state of RelA during aging to elevate Bim, DMT1, and iron (75) as well as the intracellular levels of α-synuclein (83). In turn, these events lead to α-synuclein aggregation (83), microglia activation, and neuronal damage (84). All together, these findings point to a role of c-Rel in the regulation of SNc susceptibility to aging.

Finally, latest results (unpublished results) indicate that at a premotor phase (7–12 months) when no loss of SNc DA neurons is evident yet, c-rel^−/−^ mice display olfactory deficits, gut dysfunctions, and reduced DAT immunoreactivity in the striatum. This disease progression mimics the pathological and clinical progression observed in PD patients that at premotor stage of the disease show constipation, hyposmia (85), and reduced DAT imaging by PET or SPECT scan. These findings further strengthen the notion that c-rel^−/−^ mice represent an innovative disease model suitable both for studies aimed at dissecting the mechanisms of PD onset and to test novel therapeutic approaches for intervention at the premotor stages of the disorder.

## Conclusion

Although NF-κB factors are transcriptional regulators of inflammation and apoptosis, their relevance in aging-related neurodegeneration is still underestimated.

Activation of RelA has been proposed to lead the systemic aging process in mice (86, 87), being negligible in the hypothalamus of young mice and progressively increasing, earlier in microglia and later in neuronal cells, as the mice become older (12). The genetic depletion of one allele of RelA attenuated the behavioral signs of neurodegeneration and extended the healthspan in a progeroid mouse model (13).

These stimulating results could be reread in the light of recent evidence showing that protective versus harmful p50/RelA activation strongly depends on the acetylation state of RelA. Activation of RelA subunit displaying reduced grade of general acetylation, but site-specific acetylation of K310, triggers apoptotic gene expression in brain ischemia. In the absence of such a mismatch, the activation of RelA is neuroprotective, as observed in preconditioning ischemia.

Whether a dysregulation of the RelA acetylation state is also involved in normal aging, or just in pathological aging, remains to be established. What seems promising is that aberrant RelA acetylation, more than RelA nuclear translocation, can be a suitable target. It is corrected by the synergistic association of HDAC inhibitors and resveratrol to produce neuroprotection.

While evidence suggests that RelA activation marks physiological elderly in mice, we show that the deficiency of c-Rel leads to a parkinsonian phenotype with aging (10). The degeneration of DA neurons in the SNc, the microglia activation and the α-synuclein pathology are associated with development of an l-DOPA-responsive parkinsonism. Intriguingly, in the basal ganglia of aged c-Rel deficient mice, but not in aged matched controls, the acetylation state of RelA was reminding the one observed in lethal ischemia.

This body of evidence supports the premise that the balance between c-Rel- and RelA-mediated transcription may be at the crossroad between normal and pathological aging of the mammal brain. A defect of c-Rel activity, associated with higher RelA activation, reduces SNc resilience to aging hereby leading to a late-onset form of parkinsonism. Validation of the impact of c-Rel activity in PD onset and progression is now the challenge of ongoing studies.

## Conflict of Interest Statement

The authors declare that the research was conducted in the absence of any commercial or financial relationships that could be construed as a potential conflict of interest. The Guest Associate Editor Giuseppe Pignataro declares that, despite having collaborated previously with the authors Annamaria Lanzillotta, Caterina Branca, Pier Franco Spano and Marina Pizzi, the review process was handled objectively and no conflict of interest exists.

## Abbreviations

1B/DMT1, 1B isoform of divalent metal transporter-1; AMPK, AMP-activated kinase; BDNF, brain-derived neurotrophic factor; BIRC3, baculoviral IAP repeat-containing protein 3; DA, dopaminergic; HAT, histone acetyltransferase; HDAC, histone deacetylase; K310, lysine 310; MCAO, middle cerebral artery occlusion; MnSOD, superoxide dismutase; MPP^+^, 1-methyl-4-phenylpyridinium; NAD^+^, nicotinamide adenosine dinucleotide; OGD, oxygen–glucose deprivation; PD, Parkinson’s disease; RIP1, receptor interacting protein; ROS, reactive oxygen species; SIRT1, sirtuin 1; SN, substantia nigra; SNc, SN pars compacta; TH, tyrosine hydroxylase; TNF, tumor necrosis factor; UCP4, mitochondrial uncoupling protein 4.
